# Furan‐Protected 4‐Maleimidomethyl Styrene for Reversible Crosslinked Polymers

**DOI:** 10.1002/marc.202500168

**Published:** 2025-06-23

**Authors:** Alfred Andreas Hamm, Gregor Schnakenburg, Sigurd Höger

**Affiliations:** ^1^ Kekulé‐Institut für Organische Chemie und Biochemie University of Bonn Bonn Germany; ^2^ Institut für Anorganische Chemie University of Bonn Bonn Germany

**Keywords:** Diels‐Alder reaction, maleimidomethyl styrene, self‐healing, thermoreversible crosslinking

## Abstract

Due to the growing interest in reversibly cross‐linkable polymers, the synthesis of maleimide‐containing polymers for *Diels‐Alder* crosslinking is very attractive. Here we present two new synthetic approaches to such polymers, starting from an *N*‐alkylation of furane‐protected maleimide, either at the polymeric level with poly(styrene‐*stat*‐4‐chloromethylstyrene) or at the level of monomeric structures with 4‐chloromethylstyrene. Both strategies allow freely adjustable maleimide contents and copolymerization with various comonomers. The monomer used for the second route has not yet been described in this form and is therefore examined in more detail, particularly with regard to its copolymerization behavior. We also present the reversibility of the cross‐linking reaction and the possibility of integrating further functional groups into the polymer.

## Graphical abstract

We present two new synthetic approaches to maleimide‐containing polymers for *Diels‐Alder* crosslinking, starting with the *N*‐alkylation of furane‐protected maleimide, either at the polymeric level with poly(styrene‐*stat*‐4‐chloromethylstyrene) or at the level of monomeric structures with 4‐chloromethylstyrene generating a new styrene monomer. Copolymerization parameters have been determined and the properties of the polymers and their crosslinking are described.

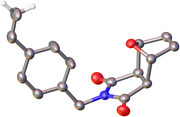

## 1 Introduction

In their diverse spectrum of applications, polymers are often exposed to high physical stress and the associated wear and tear. Whereas thermoplastics are softened above *T_g_* or *T_m_*, thermosets have often superior mechanical properties and higher stability against solvents [1, 2]. However, this goes into the costs of processing by extrusion and complicates recycling efforts [3, 4, 5]. Therefore, during the past years the interest in polymer networks with reversible covalent crosslinks has steadily increased [6, 7, 8]. Reversibility allows recycling of the covalently cross‐linked materials by bond breaking and gives access to self‐healing networks [9, 10, 11]. In this approach, linking points of the polymer network are disconnected under defined conditions, for example at elevated temperatures, and then reassembled so that occurred damages can be fixed [12, 13]. One possibility for this is cross‐linking via the *Diels‐Alder* (DA) reaction between maleimide and furan units [14, 15, 16]. This system has been described for polymers as well as for defined small molecules [17, 18, 19, 20, 21]. Whereas furfuryl methacrylate is commercially available, maleimide polymers are less common. So far, maleimide‐containing polystyrenes have been synthesized via a polymer analogs *Friedel‐Crafts*‐type alkylation of polystyrene with *N*‐chloromethyl maleimide resulting in degrees of functionalization up to about 30% [22, 23]. Here, we present two alternative pathways in which styrene‐based polymers with a freely adjustable maleimide content are easily accessible. The following crosslinking reaction can be carried out with oligo or poly furfuryl compounds e.g. poly(furfuryl methacrylate) which are already extensively described in the literature [24, 25, 26].

## 2 Results and Discussion

### 2.1 Synthesis of Maleimide‐Containing Polymers

**SCHEME 1:**
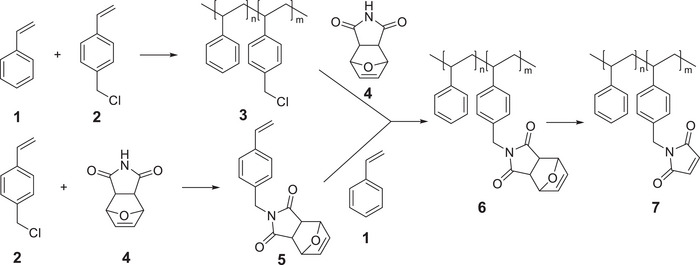
Synthetic approach toward maleimide‐containing polymers.

The syntheses of the maleimide functionalized poly(styrenes) **7** are shown in Scheme 1. Both routes have in common that we do not perform an alkylation but start with already chloromethylated styrene **2**. The first route starts with the copolymerization of styrene (**1**) with 4‐chloromethyl styrene (**2**) and subsequent substitution of the chloride of **3** (*M*_n_ = 2.3 × 10^4^ g mol^−1^; *D* = 1.7) by the protected maleimide **4**, resulting in the furan‐protected poly(styrene‐*stat*‐4‐maleimidomethylene styrene) **6** (*M*_n_ = 1.6 × 10^4^ g mol^−1^; *D* = 1.7). We have chosen **4** as a nucleophile because it is significantly more reactive toward alkyl halides than the pure maleimide [27]. This reaction can be performed at room temperature and substitution can be determined by NMR‐spectroscopy (Figure S7). Small deviations in the respective integrals are found (2.00 vs 1.94 protons, respectively) that can be attributed to measurement errors, so we assume (nearly) quantitative substitution. **4** is easily available in quantitative yield by simply mixing the diene and the dienophile in toluene at 90°C overnight. The second route starts with the nucleophilic substitution of the chloride of **2** with the protected maleimide **4** at the stage of the monomer, generating **5**. Copolymerization of **5** with **1** is an alternative way to **6** (*M*_n_ = 1.6 × 10^4^ g mol^−1^; *D* = 1.7). **6** can be thermally deprotected at 140°C to **7** by a retro‐*Diels‐Alder* reaction (Figure S11). The use of furan‐protected maleimide is absolutely crucial here, not only to increase the yield in the substitution reaction but also to prevent the maleimide double bond from participating in the polymerization [28, 29, 30]. The maleimide content is in both approaches freely variable, however, solubility problems of **6** and **7** occur if the degree of functionalization exceeds around 40%, and the homopolymer of **5** is virtually insoluble.

### 2.2 Crystal Structure, Copolymerization Parameters, and Thermal Properties of 5

**FIGURE 1:**
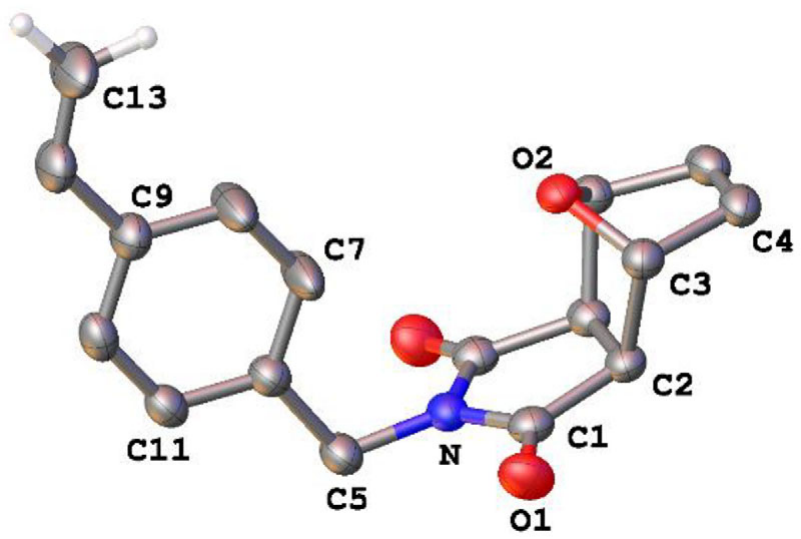
Ellipsoid plot of the molecular structure of **5** in the crystal. Thermal displacement factors are set at 50% probability. Hydrogen atoms (except those of the terminal methylene group) are omitted for clarity (selected bond lengths and angles are listed in Table S1).

Despite the high interest in thermoreversible crosslinking reactions, and although furan‐protected maleimido MMAs were reported nearly 20 years ago, we are not aware of any report about monomer **5** [31]. Single crystals of **5** were grown by slow evaporation of chloroform and confirmed the exclusive formation of the *exo* isomer of **5** under our synthetic conditions (Figure 1). In addition, the ^1^H‐NMR spectrum shows only one set of signals as also for **4** (Figures S3 and S4). It should be mentioned that the synthesis of **4** in ethyl acetate at room temperature shows the formation of more than one species, most probable *endo* and *exo* products in variable ratios [32].

In the course of the copolymerization reaction, we found that the feed ratio of the monomers is not equal to the copolymer composition. Therefore, the copolymerization behavior of **5** was investigated. Using the *Fineman‐Ross* method, the copolymerization parameters *r1* and *r2* can be determined graphically [33]. For this purpose, we performed a series of copolymerizations with different feed ratios. The copolymer composition was then determined by ^1^H‐NMR spectroscopy. This procedure was carried out for **5** and two different comonomers, 4‐methylstyrene and 4‐methoxystyrene. Monomer feed ratios, copolymer composition, and the graphical determination of *r_1_* and *r_2_* are shown in Tables S2 and S3 and Figures S20 and S22. The copolymerization parameters *r1* and *r2* were determined to be *r_1_* = 1.236 and *r_2_* = 0.771 for the copolymer **5**:4‐methylstyrene and *r_1_* = 1.480 and *r_2_* = 0.637 for the copolymer **5**:4‐methoxystyrene.

According to *Alfrey* and *Price*, the monomer‐specific parameters *Q* and *e* can be determined from the copolymerization parameters [34]. This can be used to estimate how well two monomers polymerize with each other. Monomers with different *Q* values copolymerize poorly whereas ideal copolymerization is found for monomers with similar *Q* and *e* values [35]. As the *Q* and *e* values are relative values, the values for the comonomer have already been known and taken from the literature [36]. With the reference *Q* and *e* values for 4‐methylstyrene and 4‐methoxystyrene, we determined for **5** the value for *Q* as 1.64 and for *e* as −0.85, and *Q* as 1.63 and for *e* as −0.87, respectively (Table 1).

**TABLE 1:** Reference *Q‐e*‐values of 4‐methylstyrene and 4‐methoxystyrene and calculated *Q‐e*‐values of **5** [36].

Comonomer	*Q,e*‐values of comonomer	Calculated *Q,e*‐values of 5
4‐Methylstyrene	*Q* = 1.10, *e* = −0.63	*Q* = 1.64, *e* = −0.85
4‐Methoxystyrene	*Q* = 1.36, *e* = −1.11	*Q* = 1.63, *e* = −0.87

The values we obtained are consistent with the observation that copolymerization of **5** and acrylates or methacrylates is rather difficult and sometimes yields low conversions and mixing ratios in the polymer, which vary greatly from the ratios in the reaction mixture.

**FIGURE 2:**
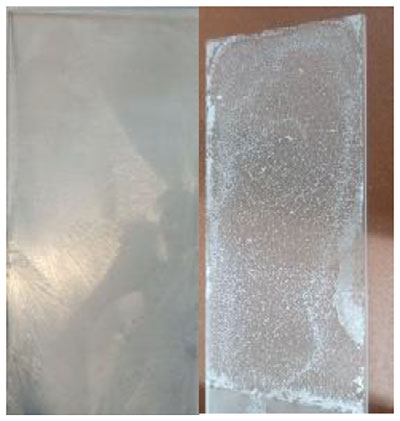
(a) **5** on a slide from a toluene solution dried at 80°C, (b) and after heating to 150°C.

When neat samples of **5** are heated, a clear morphological change of the substance can be observed around 140°C, where the colorless, loose powder turned to a pale yellow, compact, and hard solid. However, a melting point could not be determined. From approximately 230°C up to the maximum temperature of the measuring device used of 260°C, the solid slowly began to darken without melting. One possible explanation for the morphological change is the cleavage of furan in the course of a retro‐*Diels‐Alder* reaction, as this also takes place in the case of polymers at around this temperature range. The absence of a detectable melting point indicates a thermal crosslinking of the material [37, 38]. When a bulk sample of **5** was heated to 150°C for 1.5 h, a weight‐loss of around 14% was observed, indicating an incomplete retro‐DAreaction, as compared to a theoretical weight‐loss of 24% for a complete furan loss. In addition, the obtained material is completely insoluble in all common solvents which stands in sharp contrast to the highly soluble **5**. When a solution of **5** in toluene was evaporated on a glass slide and dried at 80°C overnight (Figure 2), a crystalline appearing film was formed that changed its color to slightly yellow after being heated to 150°C overnight. The resulting brittle and rough surface has a low adhesion force and is therefore hydrophobic and applied water droplets quickly roll off.

Contrary, the thermogravimetric analysis (TGA) measurement of poly(styrene‐stat‐**5**) (**6**) showed a weight loss of around 6.7% in a temperature window between 142°C and 187°C (Figure S10). This is in good correlation with the NMR spectrum of the polymer used, which showed a monomer ratio of styrene to **5** of about 3.6 to 1. Thus, the observed weight loss is consistent with a complete retro‐*Diels‐Alder*. During the TGA measurement, the polymer then remains stable up to around 350°C before thermal degradation occurs.

**SCHEME 2:**
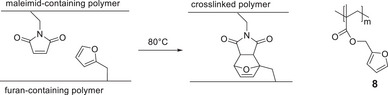
Crosslinking of the maleimide and the furyl functionalized polymers and structure of **8**.

### 2.3 Crosslinking, Self Healing and Functionalization of 7

The furan protection group can be removed from **6** by heating a solution of the polymer in 1,1,2,2‐tetrachloroethane at 140°C overnight. The completeness of this deprotection was confirmed via ^1^H‐NMR‐spectroscopy (Figure S11). A solution of the resulting polymer **7** in toluene was mixed with a hot solution of poly(furfuryl methacrylate) (**8**) in toluene in equimolar amounts of the functional groups, and a glass slide was cast and dried at 80°C. A slightly orange, homogeneous brittle film was obtained, which was completely insoluble, indicating at least partial crosslinking (Scheme 2). IR spectra of the polymers are not significant due to band overlapping. However, the disappearance of the glass transition temperature of **8** in the mixed polymer was an indication of the crosslinking as well as the insolubility of the formed film. Unfortunately, the healing of small surface defects at 150°C could not be observed.

**FIGURE 3:**
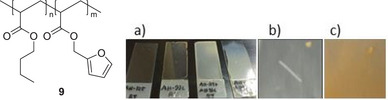
left: Structure of **9**; right: (a) from left to right: Film of pure poly(butyl acrylate‐*stat*‐furfuryl methacrylate), film of pure **7**, film of equimolar mixtures of both polymers, dried at room temperature, film of equimolar mixtures of both polymers, dried at 80°C, (b) scratch in 80°C‐film, (c) disappeared scratch after heating to 150°C and appearance of brownish‐yellow color.

Therefore, we have chosen poly(butyl acrylate‐*stat*‐furfuryl methacrylate) (**9**) as the diene source which is also much better soluble in toluene. While the toluene solutions of **7** as well as **9** were separately cast on glass slides, clear, homogeneous films were obtained after drying. When, on the other hand, a mixture of both polymers with equimolar amounts of functional groups in toluene was cast onto a glass slide, a homogenous but opaque film was formed at room temperature as well as at 80°C (Figure 3). While both films **7** and **9** are partly soluble in dichloromethane, the cross‐linked film from the polymer mixture was insoluble in dichloromethane but peeled off after some time. To test the reversibility of the crosslinking, a small scratch was made on the surface and the film was heated to 150°C. After 24 h, the scratches had almost completely disappeared. This is in good agreement with the result of the TGA analysis showing that the retro‐*Diels‐Alder* reaction for polymer **6** takes place at a temperature above 142°C. However, it should be noted that the films obtained a brownish‐yellow color which did not disappear even after cooling.

**SCHEME 3:**
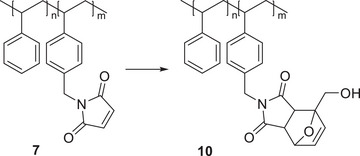
Functionalization of **7** with furfuryl alcohol to yield **10**.

In addition to the possibility of using the maleimide group to crosslink the polymer, it is also possible to functionalize the polymer via this group using a *Diels‐Alder* reaction so that further functional groups can be incorporated into the polymer. For example, with furfuryl alcohol (Scheme 3), this allows the incorporation of hydroxyl groups, as in **10**. Furthermore, it should also be possible to combine crosslinking and functionalization in the case of only partial functionalization.

## 3 Conclusion

In summary, we described a new styrene monomer with a pendant protected maleimide group, which was investigated with regard to its copolymerization behavior and its thermal properties. This monomer can be used for a new synthetic approach for the synthesis of thermoreversible crosslinked polymers. By means of simple copolymerization, maleimide‐containing copolymers of various compositions and variable adjustable maleimide content can be obtained. In addition, we were able to present an alternative synthetic route toward maleimide‐containing copolymers via the *N*‐alkylation of protected maleimide with poly(styrene‐*stat*‐4‐chloromethylstyrene). Thermal deprotection gave maleimide‐containing polymers that were crosslinked with poly(butyl acrylate‐*stat*‐furfuryl methacrylate) using the *Diels‐Alder* reaction. The reversibility of this crosslinking and the resulting self‐healing properties were demonstrated. Furthermore, we were able to show that it is also possible to functionalize the maleimide groups. Based on these findings, the possibility of combining functionalization and cross‐linking will be investigated in the future. In addition, it is to avoid or reduce the orange coloring of the films when heated.

## Conflicts of Interest

The authors declare no conflicts of interest.

## Supporting information




**Supporting File 1**: marc202500168‐sup‐0001‐SuppMat.pdf

**Supporting File 2**: marc202500168‐sup‐0002‐SuppMat.cif

## Data Availability

The data that support the findings of this study are available in the supplementary material of this article.
