# Supplementary material for: Furan‐Protected 4‐Maleimidomethyl Styrene for Reversible Crosslinked Polymers
Source: Macromol Rapid Commun. 2025 Jun 23;46(23):e00168. doi: 10.1002/marc.202500168 (PMC12687677; doi:10.1002/marc.202500168)
Supplement: Supplementary file 1 — Supporting File 1: marc202500168‐sup‐0001‐SuppMat.pdf [file MARC-46-e00168-s001.pdf]

Furan-Protected 4-Maleimidomethyl Styrene for Reversible Crosslinked Polymers

Alfred Andreas Hamm, Gregor Schnakenburg, and Sigurd Höger*

A. A. Hamm, S. Höger

Kekulé-Institut für Organische Chemie und Biochemie

University of Bonn

Gerhard-Domagk-Str. 1

53123 Bonn

E-Mail: hoeger@uni-bonn.de

G. Schnakenburg

Institut für Anorganische Chemie

University of Bonn

Gerhard-Domagk-Str. 1

53123 Bonn

Supporting Information

Content

**1. General**	**3**
**2. Analytical Methods**	**3**
**3. Synthesis and Copolymerization Parameters**	**4**

1. General

Unless otherwise stated, all reactions were carried out under ambient conditions. Reactions carried out under inert gas conditions were performed under standard *Schlenk* conditions with argon N46 (purity ≥ 99.996%, H_2O content <10 ppm, O_2 content <6 ppm, N_2 content < 20 ppm) from Air Liquid. The reaction vessels of such reactions were sealed with solid rubber septa and connected to an oil-filled pressure relief valve. The reactions were carried out in borosilicate glass vessels of different volumes and ground joint sizes. Styrene, 4-chloromethylstyrene, 4-methylstyrene, 4-methoxystyrene, furfuryl methacrylate, butyl acrylate and dichloromethane (DCM) were freshly distilled before used, all other chemicals were used as purchased.

2. Analytical Methods

NMR-Spectroscopy

All samples for ^{1}H- and ^{13}C-NMR spectroscopy were prepared under ambient conditions. Spectra were measured with the Bruker Avance I 400 MHz, Bruker Avance I 500 MHz, Bruker Avance III HD 500 MHz Prodigy and Bruker Avance III HD 700 MHz Cryo spectrometers from Bruker. The spectra were analysed using the programmes MestReNova 8.01 and MestReNova 14.2.1 from Mestrelab Research. Tetramethylsilane was used as a reference standard for the calibration of the solvent signals. All spectra were referenced to the signal of the deuterated solvent.

Mass Spectrometry

All mass spectra were prepared under ambient conditions and measured on either the Orbitrap XL spectrometer from Thermo Fisher Scientific, the MAT 95 XL spectrometer from Thermo Finnigan or the micrOTOF-Q spectrometer from Bruker Daltonic.

TGA

TGA measurements were carried out with the TGA/SDTA851^{e} instrument from Mettler Toledo. The experiments were carried out under nitrogen atmosphere in an aluminium sample crucible with a heating rate of 10 °C/min in a temperature range from 100 °C to 550 °C. The measurements were analysed using STAR^{e} SW 9.01 software from Mettler Toledo.

DSC

All DSC measurements were carried out with the DCS823^{e} instrument from Mettler Toledo. The experiments were carried out in aluminium sample crucible under a nitrogen atmosphere with a gas flow rate of 100 mL/min and a heating rate of 10 °C/min. The samples were analysed in a temperature range of 20-170 °C, whereby two heating and cooling cycles were run through in each case. The measurements were analysed using the STAR^{e} SW 18.00 program from Mettler Toledo and the second heating curve was used for analysis for all experiments.

Gel Permeation Chromatography (GPC)

Molecular weights where determined by using an analytical GPC from Agilent Technologies with the following configurations: Pump: IsoPump G1310A; Autosampler: ALS G1329A; UV-Detector: VWD G1314B: RI-Detector; RID G1362A; Columns: 4 columns from PSS Polymer Standard Service GmbH, Mainz, Germany; SDV, 8 mm × 300 mm, porosity: 10^{2} Å, 10^{3} Å, 10^{5} Å, 10^{6} Å, with precolumn; calibration: Polystyrene-Standards (PSS Polymer Standard Service GmbH, Mainz, Germany)
The flowrate during the measurement was 1 ml*min^{-1}. Solvent: THF from the company VWR, stabilized with 250 ppm BHT.

Single Crystal X-Ray Analysis

Data collected on a STOE STADIVARI diffractometer at 150 K (Oxford Cryostream 800er series).

3. Synthesis and Copolymerization Parameters

3

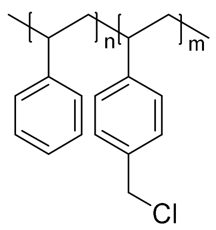

Styrene (2.730 g, 26.21 mmol, 3.07 eq.) and 4-chloromethylstyrene (1.408 g, 8.525 mmol, 1.00 eq.) were dissolved in toluene (26 mL) and the solution was flushed with argon for 30 min at room temperature. The solution was heated to 65 °C under stirring and AIBN (10 mg) in toluene (3 mL) was added. After 3 h and again after 20 h, AIBN (4 mg) in toluene (1.5 mL) was addad. After 44 h, the reaction solution was allowed to cool to room temperature and was concentrated under reduced pressure, until only a small amount of solvent remained. The remaining solution was poured into an excess of methanol, resulting in the precipitation of a colourless solid. This was sedimented using a centrifuge, washed with methanol and transferred to a round bottom flask with DCM. The solvent was removed under reduced pressure and the product was dried under vacuum until weight constant was received. **3** was obtained as a colourless solid in 37% yield (1.536 g, 4.159 mmol).
^{1}H-NMR (499.1 MHz, $CDCl_3$, 298 K, ppm) δ = 7.25-6.85 (m), 6.85-6.15 (m), 4.61-4.39 (m), 2.32-1.01 (m). From the NMR signals a ratio n:m can be estimated to be in the order of 2:1.
GPC: (PS calibration) $M_n = 3.8*10^4$, Đ = 1.7.

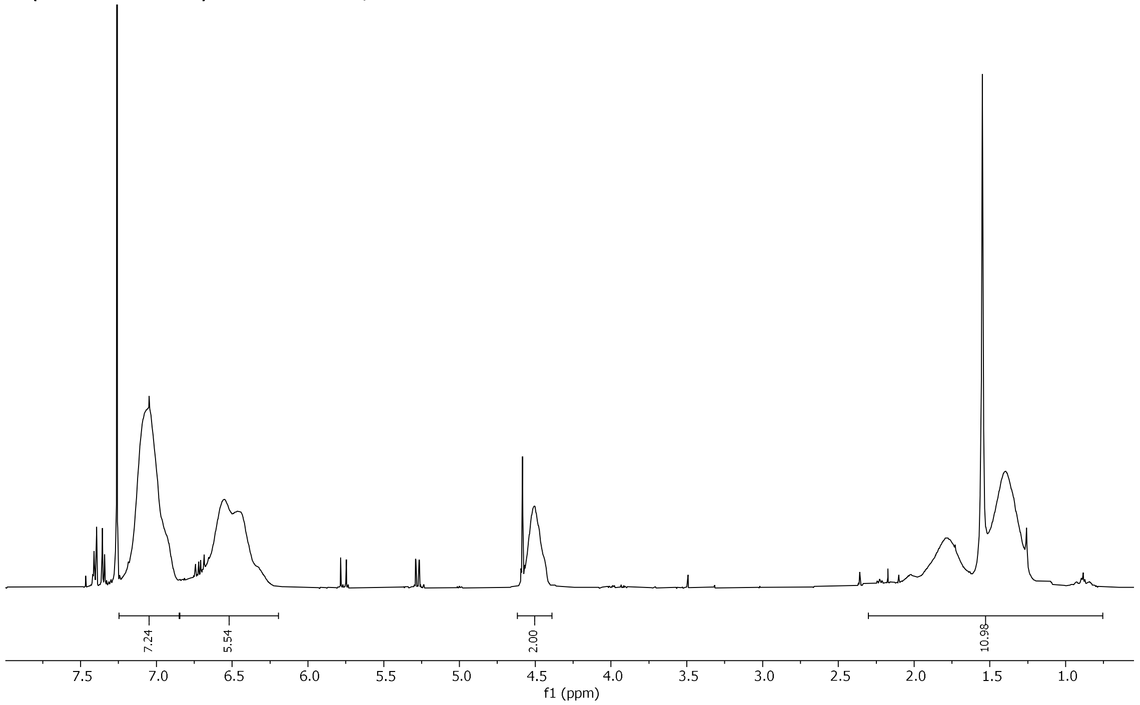

Supplementary figure S1 ^{1}H-NMR of **3**.

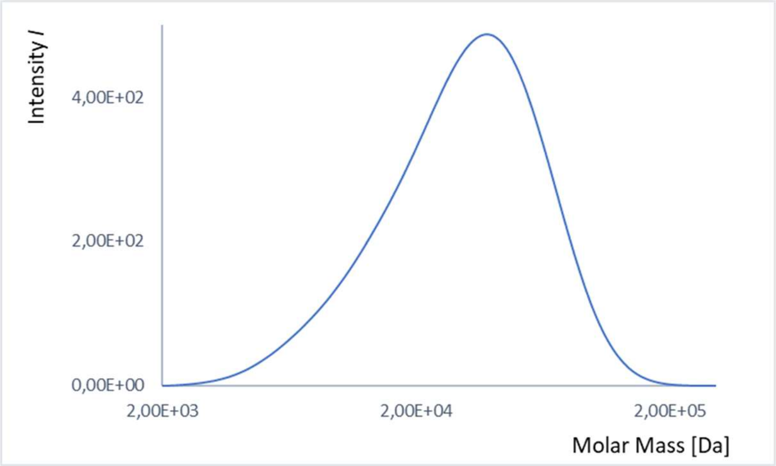

Supplementary figure S2 GPC-elugram of **3** vs PS calibration.

4

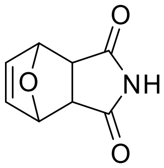

Maleimide (20.00 g, 206.0 mmol, 1.00 eq) was suspended in toluene (128 mL) and heated to 60 °C. The mixture was stirred for 10 min until a clear solution was obtained. Furan (30 mL, 28.20 g, 414.2 mmol) was then added, the reaction vessel was fitted with a reflux condenser and the mixture was heated to 90 °C. The initially pale yellow solution was stirred for 20 h, during which time a colourless solid precipitated. The solid was filtered off, washed with diethyl ether, vacuum filtered for 30 min and transferred to a round bottom flask and dried in vacuum. **4** was obtained as a colourless solid in 97% yield (33.08 g, 200.3 mmol).

^{1}H-NMR (500.0 MHz, DMSO-d_6, 298 K, ppm) δ = 11.16 (bs, 1H), 6.55 (t, $^3J_{H,H}$ = 0.9 Hz, 2H), 5.13 (t, $^3J_{H,H}$ = 0.9 Hz, 2H), 2.87 (s, 2H). **^{13}C{H}-NMR** (125.7 MHz, DMSO-d_6, 298 K), δ [ppm] = 177.9, 136.5, 80.3, 48.4, 48.4. **EI**: *m/z* (rel. int. [%]) = 68.0 (100), 97.0 (40), 121.0 (10), 138.0 (19), 165.0 (14), calculated: 165.04 g*mol^{-1}.

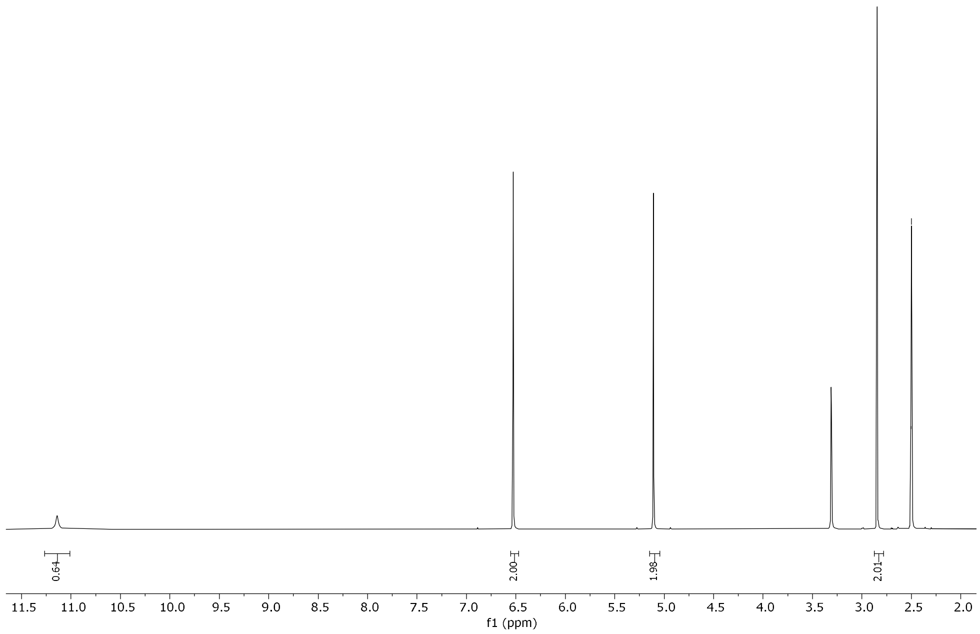

Supplementary figure S3 ^{1}H-NMR of **4**.

5

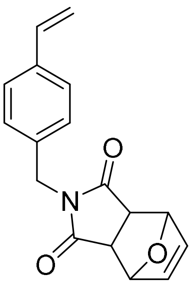

4 (5.381 g, 32.58 mmol, 1.00 eq) and K_2CO_3 (5.404 g, 39.10 mmol, 1.20 eq) were dissolved in DMF (200 mL) and stirred for 20 min at room temperature. Then, 4-chloromethylstyrene (4.973 g, 32.58 mmol, 1.00 eq) was added, resulting in an instant colour change from colourless to yellow. The reaction mixture was stirred for 22 h at room temperature. The solution was poured into an excess of water. A solid precipitated, which was filtered off and washed with water. **5** was obtained as a colourless solid in 98% yield (9.018 g, 32.06 mmol).

^{1}H-NMR (499.1 MHz, $CDCl_3$, 298 K, ppm) δ = 7.39-7.27 (m, 4H), 6.67 (dd, $^{3}J_{H,H}$ = 17.6, $^{3}J_{H,H}$ = 10.9 Hz, 1H), 6.51 (t, $^{3}J_{H,H}$ = 1.0 Hz, 2H), 5.71 (dd, $^{3}J_{H,H}$ = 17.6, $^{2}J_{H,H}$ = 0.9 Hz, 1H), 5.29 (t, $^{3}J_{H,H}$ = 1.0 Hz, 2H), 5.22 (dd, $^{3}J_{H,H}$ = 10.8, $^{2}J_{H,H}$ = 0.9 Hz, 1H), 4.63 (s, 2H), 2.86 (s, 2H). **^{13}C{H}-NMR** (125.7 MHz, $CDCl_3$, 298 K), δ [ppm] = 176.0, 137.3, 136.7, 136.5, 135.1, 128.6, 126.6, 114.3, 81.1, 47.7, 42.4. **APCI**: *m/z* (rel. int. [%]) = 1117.070 (91), 214.086 (100), 282.086 (30), calculated: 281.11 g*mol^{-1}.

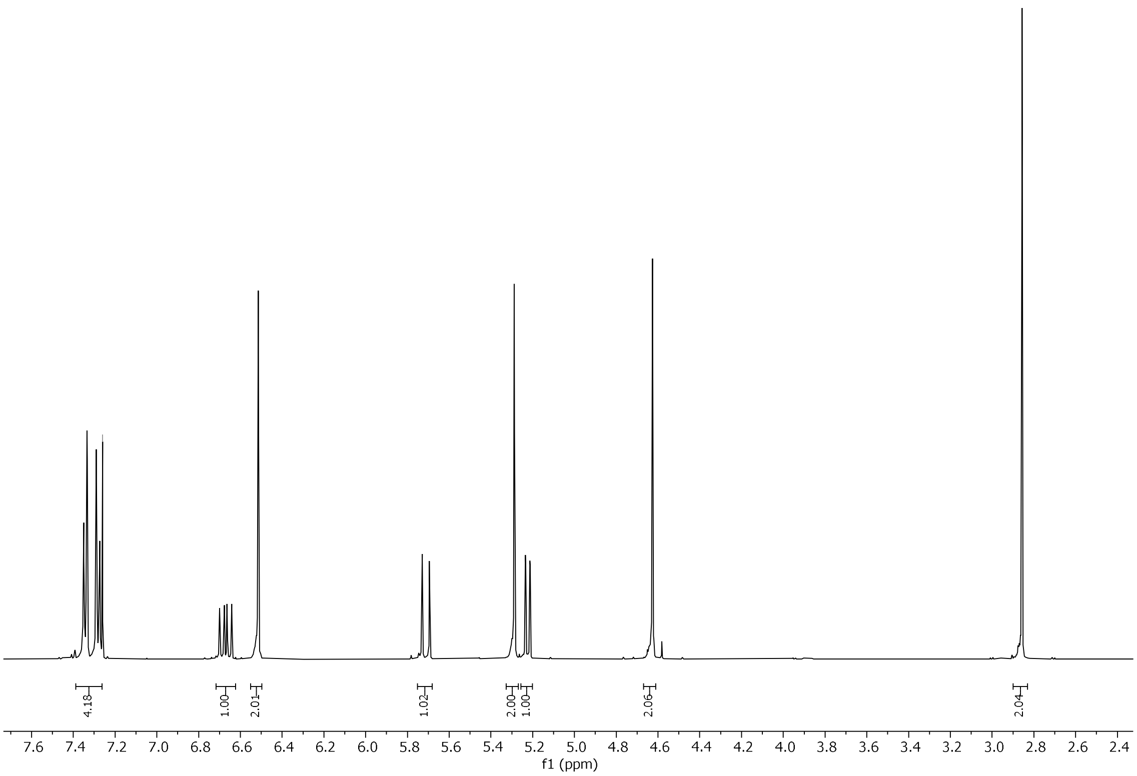

Supplementary figure S4 ^{1}H-NMR of **5**.

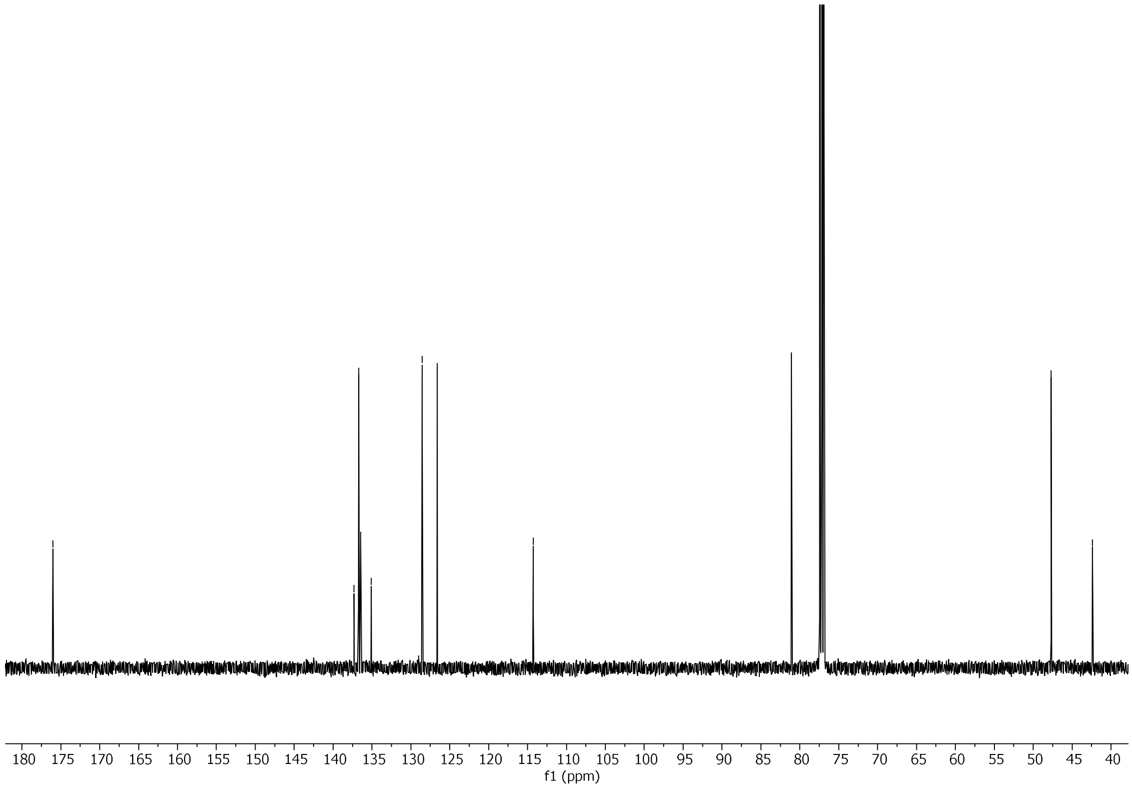

Supplementary figure S5 ^{13}C-NMR of **5**.

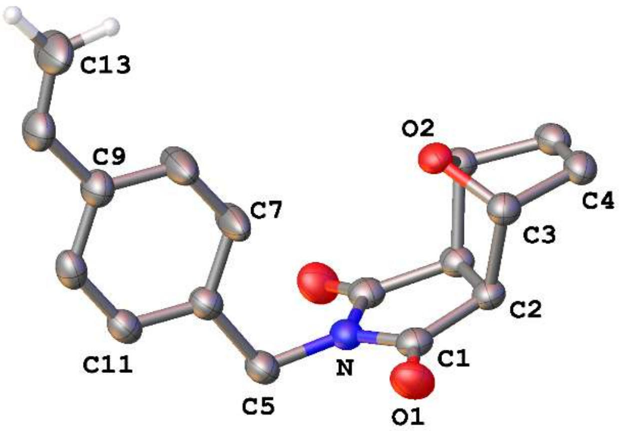

Supplementary figure S6 Ellipsoid plot of the molecular structure of **5** in the crystal. Thermal displacement factors are set at 50% probability. Hydrogen atoms (except those of the terminal methylene group) are omitted for clarity.

Data of a clear colourless plate like specimen (0.40×0.25×0.10 mm^3) of **5** were collected on a STOE STADIVARI diffractometer at 150 K (Oxford Cryostream 800er series). Crystal data for $C_{17}H_{15}NO_3$: Orthorhombic, space group *Pnma*, a = 10.4267(4) Å, b = 8.4349(4) Å, c = 15.6093(6) Å, V = 1372.81(10) $Å^3$ (Z = 4), ρ_{calc} = 1.361 gcm^{-3}, $2\theta_{max}$ = 141°, Cu-K_α (λ = 1.54186 Å), 12796/1398 collected/independent reflections (R_{int} = 0.0435, R_{sigma} = 0.0203), 1398/0/116 data/restraints/parameters, GOOF = 1.045, R_1 = 0.0438, wR_2 = 0.1265 (I > 2s(I)); R_1 = 0.0501, wR_2 = 0.1342 (all data); intrinsic phasing methods (SHELXT program system: G. M. Sheldrick, *Acta Crystallogr. A Found. Adv.*, **2015**, *71*, 1, 3-8), refined by full matrix least-squares/difference Fourier synthesis with ShelXL-2019/3 (G. M. Sheldrick, *Acta Crystallogr. C*, **2015** *71*, 1, 3-8). CCDC number 2423287 contains the supplementary crystallographic data for this paper, which can be obtained free of charge from the Cambridge Crystallographic Data Centre via http://www.ccdc.cam.ac.uk/data request/cif.

Supplementary table S1. Bond Lengths and Angles for **5**.

Atom	Atom	Length/Å	Atom	Atom	Length/Å	Atom	Atom	Atom	Angle/°	Atom	Atom	Atom	Angle/°
O1	C1	1.2080(18)	C4	C4[1]	1.323(4)	C3[1]	O2	C3	96.08(13)	C4[1]	C4	C3	105.65(9)
O2	C3	1.4401(16)	C5	C6	1.520(3)	C1[1]	N	C1	113.08(16)	N	C5	C6	112.90(15)
O2	C3[1]	1.4401(16)	C6	C7	1.388(3)	C1	N	C5	123.46(8)	C7	C6	C5	123.18(19)
N	C1	1.3836(17)	C6	C11	1.384(3)	C1[1]	N	C5	123.46(8)	C11	C6	C5	118.88(17)
N	C1[1]	1.3836(17)	C7	C8	1.381(3)	O1	C1	N	124.35(14)	C11	C6	C7	117.95(19)
N	C5	1.455(2)	C8	C9	1.389(3)	O1	C1	C2	127.08(14)	C8	C7	C6	121.0(2)
C1	C2	1.512(2)	C9	C10	1.385(3)	N	C1	C2	108.56(12)	C7	C8	C9	121.6(2)
C2	C2[1]	1.538(3)	C9	C12	1.475(3)	C1	C2	C2[1]	104.76(8)	C8	C9	C12	123.0(2)
C2	C3	1.5681(18)	C10	C11	1.387(3)	C1	C2	C3	111.03(11)	C10	C9	C8	116.83(19)
C3	C4	1.5172(19)	C12	C13	1.299(4)	C2[1]	C2	C3	101.10(8)	C10	C9	C12	120.15(19)
						O2	C3	C2	100.87(11)	C9	C10	C11	122.09(19)
						O2	C3	C4	101.99(12)	C6	C11	C10	120.50(18)
						C4	C3	C2	105.99(11)	C13	C12	C9	127.3(2)

[1] +X,1/2-Y,+Z

6 - Variant A

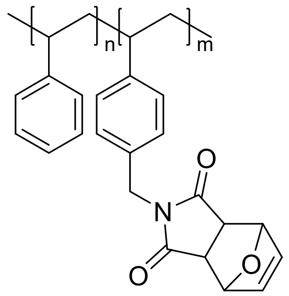

4 (0.640 g, 4.193 mmol, 1.01 eq) and K_2CO_3 (0.642 mg, 4.647 mmol, 1.12 eq) were dissolved in DMF (60 mL) and stirred for 15 min at room temperature. Subsequently, **3** (1.536 g, 4.159 mmol, 1.00 eq) was added and the mixture was stirred for 24 h at room temperature. After this, the reaction mixture was poured into ice water. A colourless solid precipitated, which was filtered off and washed with water. The crude product was suspended in MeOH and refluxed for 2.5 h. After cooling to room temperature, the solid was filtered off, transferred to a flask and dried to constant weight under reduced pressure. **6** was obtained as a colourless solid in quantitative yield.

^{1}H-NMR (499.1 MHz, $CDCl_3$, 298 K, ppm) δ = 7.30-6.78 (m), 6.78-6.01 (m), 5.36-5.15 (m), 4.68-4.40 (m), 2.93-2.62 (m), 2.37-0.80 (m).

GPC: (PS calibration) $M_n = 1.6*10^4$, Đ = 1.7.

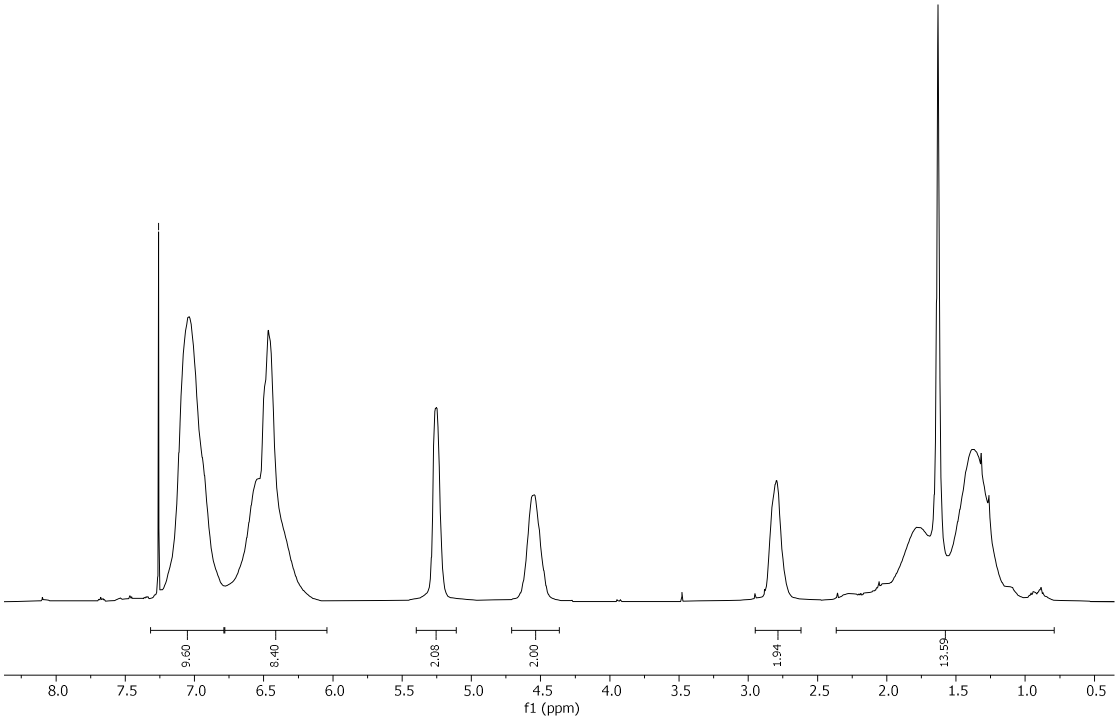

Supplementary figure S7 ^{1}H-NMR of **6 - variant A**.

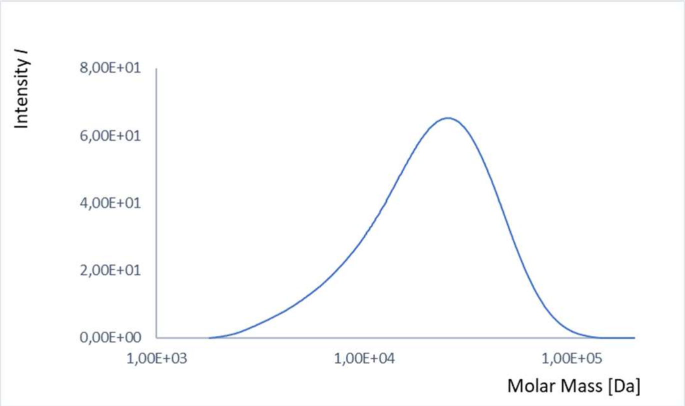

Supplementary figure S8 GPC-elugram of **6** vs PS calibration **variant A**.

6 - Variant B

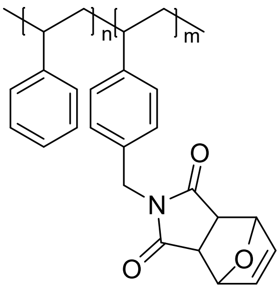

5 (946.2 mg, 3.366 mmol, 1.00 eq) was suspended in toluene (15 mL), heated to 65 °C and stirred until a clear solution was obtained. Styrene (1.856 g, 17.82 mmol, 5.30 eq) was added and the reaction mixture was flushed with argon for 30 min. AIBN (10 mg) in toluene (1 mL) was added and the mixture was stirred for 18.5 h at 65 °C. After this, again AIBN (8 mg) in toluene (1 mL) was added and the mixture was stirred for another 2 h. After cooling to room temperature, the reaction mixture was poured into an excess of ethanol. A colourless precipitate was formed, which was filtered off and then dissolved in a small amount of DCM and precipitated again by adding ethanol. The solid was filtered off and washed with ethanol. **6** was obtained as a colourless solid in 39% yield (1.090 g, 1.481 mmol).
^{1}H-NMR (400.1 MHz, $CDCl_3$, 298 K, ppm) δ = 8.13-5.63 (m), 5.42-5.10 (m), 4.71-4.36 (m), 2.97-2.62 (m), 2.48-0.60 (m). From the NMR signals a ratio n:m can be estimated to be in the order of 3.7:1.
GPC: (PS calibration) $M_n = 9.7*10^3$, Đ = 1.8.

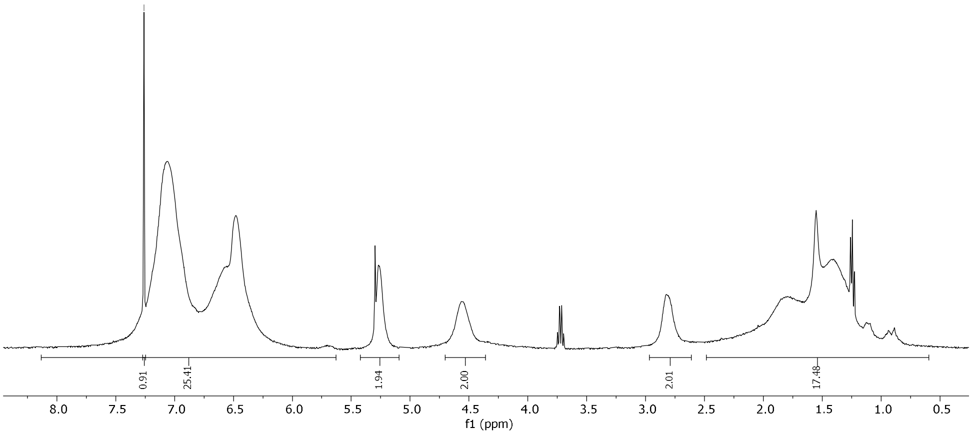

Supplementary figure S9 ^{1}H-NMR of **6 – variant B**.

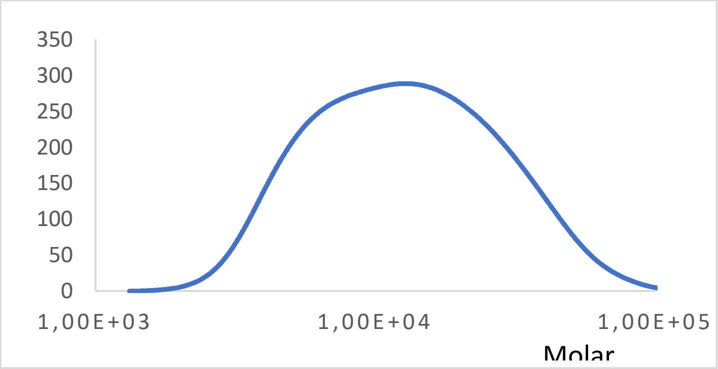

Supplementary figure S10 GPC-elugram of **6** vs PS calibration **variant B.**

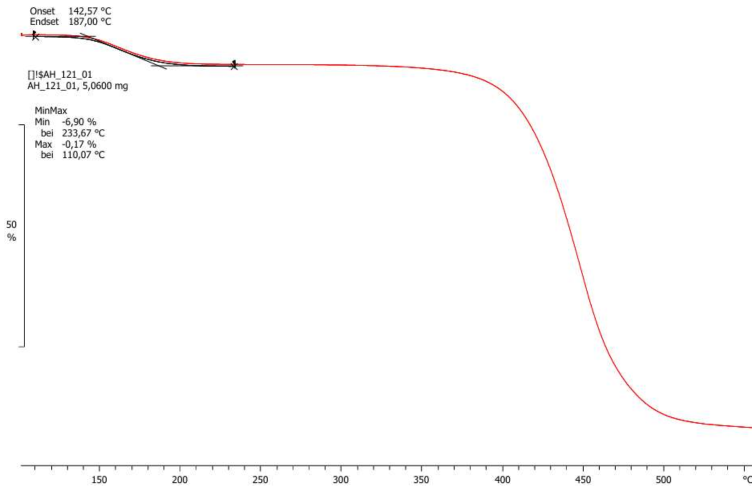

Supplementary figure S11 TGA-Measurement of **6**.

7

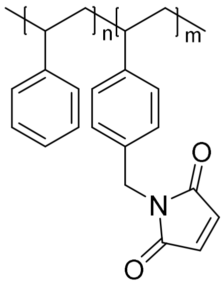

6 (from variant A; 2.049 g, 4.159 mmol) was dissolved in 1,1,2,2-tetrachloroethane (40 mL). The mixture was heated to 140 °C and stirred in an open reaction vessel for 23 h. After cooling to room temperature, the solvent was removed under reduced pressure and the product was dried to constant weight. **7** was obtained as an orange, foamy solid in quantitative yield.
^{1}H-NMR (500.2 MHz, $CDCl_3$, 298 K, ppm) δ = 7.24-6.11 (m), 4.74-4.34 (m), 2.17-0.79 (m). **^{13}C{H}-NMR** (125.8 MHz, $CDCl_3$, 298 K), δ [ppm] = 170.6, 145.3, 134.3, 128.1, 125.9, 41.3, 40.5.
GPC: (PS calibration) $M_n = 1.6*10^4$, Đ = 1.7.

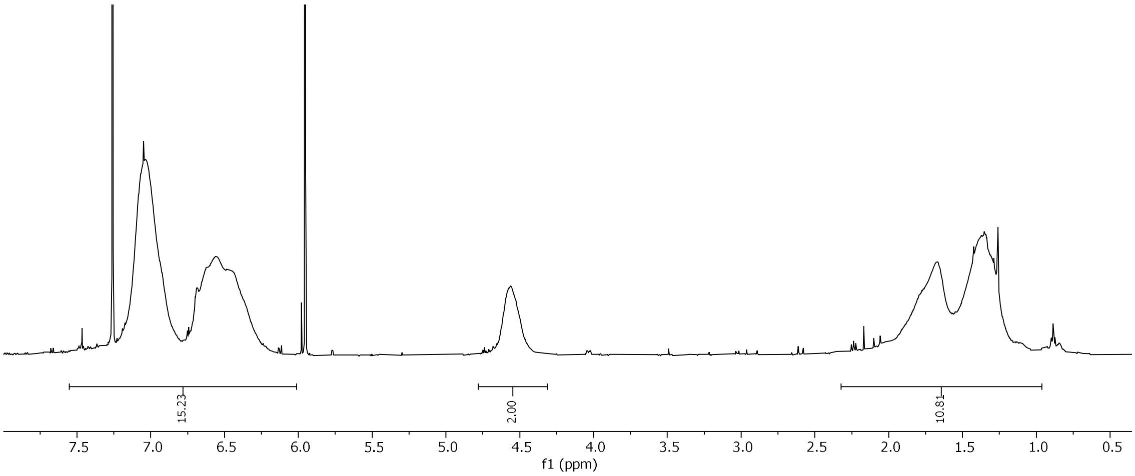

Supplementary figure S12 ^{1}H-NMR of **7**.

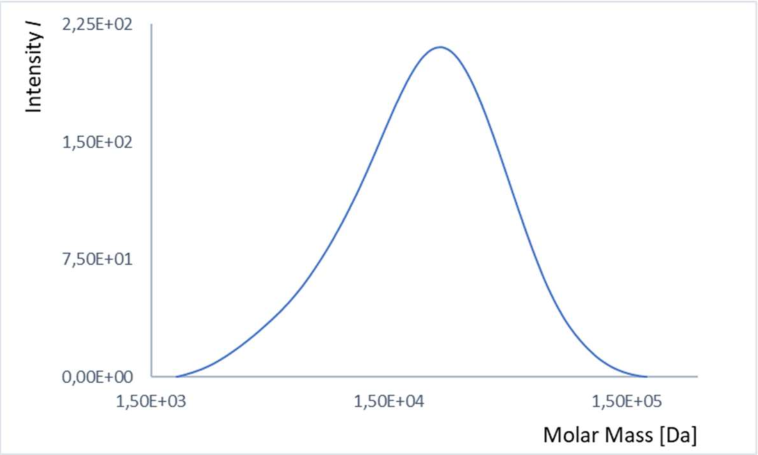

Supplementary figure S13 GPC-elugram of **7** vs PS calibration.

8

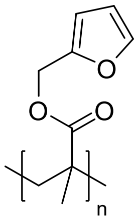

This reaction was carried out under inert gas. Under argon, toluene (23 mL) was introduced into a previously flame dried *Schlenk* flask. Furfuryl methacrylate (3.304 g, 19.89 mmol) was added and the reaction solution was flushed with argon for 30 min. The reaction solution was heated to 65 °C, then AIBN (9.7 mg) in toluene (1 mL) was while stirring. After 4 h, another portion of AIBN (4.5 mg) in toluene (1 mL) was added and the resulting mixture was stirred for a further 18 h at 65 °C. After cooling to room temperature, the solvent was removed under reduced pressure and the resulting solid was dried to constant weight. **8** was obtained as a colourless, transparent solid in quantitative yield (3.298 g, 19.85 mmol). Due to its restricted solubility in THF and chloroform, a GPC could not be recorded.
^{1}H-NMR (700.4 MHz, Toluene-d_8, 298 K, ppm) δ = 7.24-7.12 (m, 1H), 6.41-6.18 (m, 1H), 6.18-6.01 (m, 1H), 5.13-4.83 (m, 2H), 2.32-0.95 (m, 5H). **^{13}C{H}-NMR** (176.1 MHz, Toluene-d_8, 298 K), δ [ppm] = 177.3, 150.2, 143.8, 111.6, 111.1, 58.9, 45.9, 30,7, 17.1.

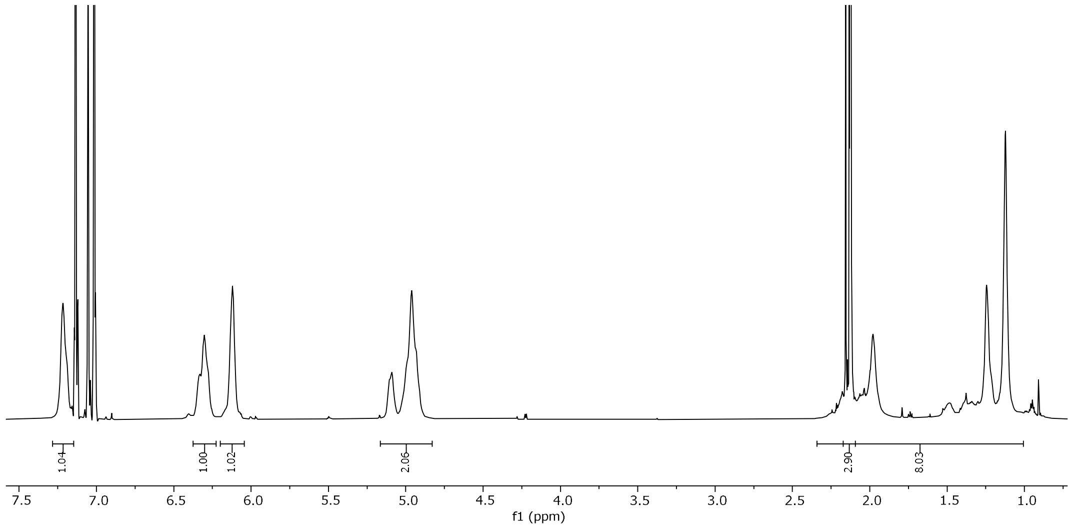

Supplementary figure S14 ^{1}H-NMR of **8**.

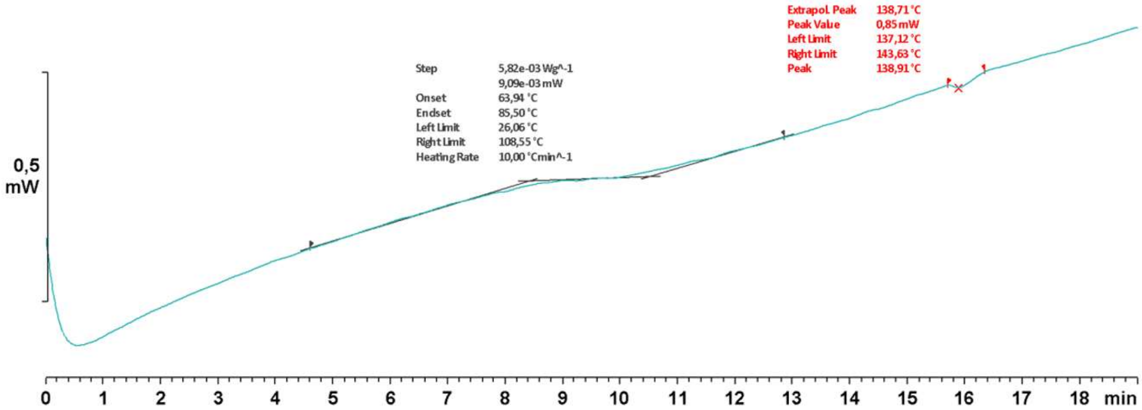

Supplementary figure S15 DSC-Measurement of **8** with glass transition at approximately 75 °C.

9

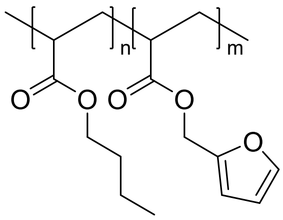

Furfuryl methacrylate (1.078 g, 6.487 mmol, 1.00 eq) and butyl acrylate (6.300 g, 49.15 mmol, 7.58 eq) were dissolved in toluene (30 mL) and the solution was flushed with argon for 30 min at room temperature. The mixture was then heated to 65 °C and AIBN (10 mg) was added as a solution in toluene (2 mL). The resulting reaction mixture was stirred for 40.5 h at 65 °C. After cooling to room temperature, the reaction mixture was poured into an excess of methanol. The result was a turbid suspension, which was sedimented using a centrifuge. The solution was decanted and the resulting highly viscous liquid was washed with methanol. The crude product obtained was dissolved twice in a small amount of DCM, precipitated by adding an excess of methanol and isolated as described before. The product was dried under reduced pressure until it reached constant weight. **9** was obtained as a

colourless, transparent, sticky solid in 36% yield (2.682 g, 3.537 mmol). Due to its restricted solubility in THF and chloroform, a GPC could not be recorded

^{1}H-NMR (700.4 MHz, $CDCl_3$, 298 K, ppm) δ = 7.50-7.35 (m), 6.51-6.28 (m), 5.22-4.76 (m), 4.29-3.71 (m), 2.73-0.45. From the NMR signals a ratio n:m can be estimated to be in the order of 1:4.6. **^{13}C{H}-NMR** (125.8 MHz, $CDCl_3$, 298 K), δ [ppm] = 175.5, 174.7, 143.4, 110.7, 110.7, 64.6, 58.5, 41.5, 30.8, 30.8, 30.7, 19.3, 13.9.

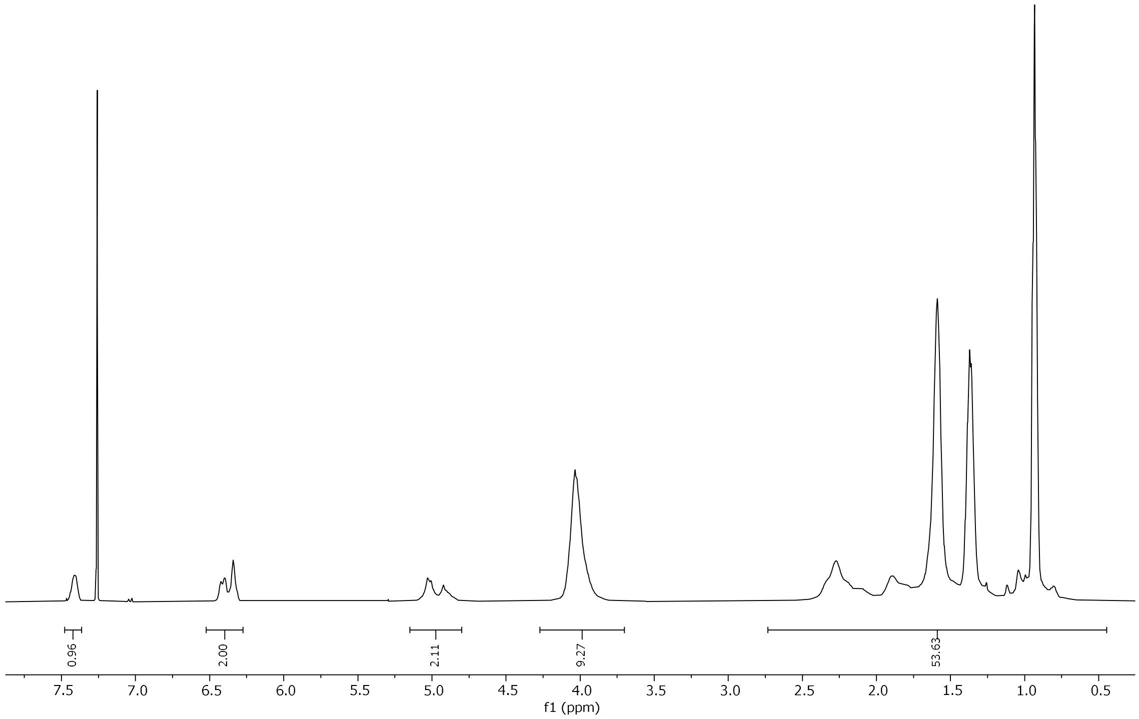

Supplementary figure S16 ^{1}H-NMR of **9**.

10

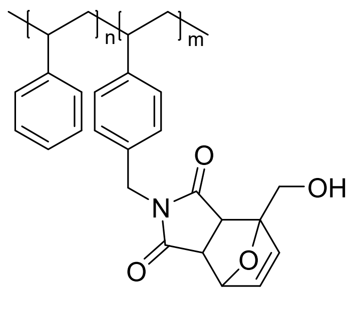

7 (480.7 mg, 1.152 mmol, 1.00 eq) was dissolved in toluene (16 mL). Furfuryl alcohol (113.0 mg, 1.152 mmol, 1.00 eq) was added, the reaction mixture was heated to 100 °C and stirred for 24 h. After cooling to room temperature, the reaction mixture was poured into an excess of methanol. An orange-brown solid precipitated, which was separated using a centrifuge. The solution was decanted and the solid was washed with methanol. The crude product was dissolved in a small amount of DCM and precipitated again with an excess of methanol. After separation of the solid using a centrifuge and washing with methanol, the solid was transferred to a round bottom flask and dried under reduced pressure until constant weight was achieved. **10** was obtained in 60% yield (based on the amount of furfuryl acohol) (409.1 mg) as an orange-brown solid.

^{1}H-NMR (500.2 MHz, $CDCl_3$, 298 K, ppm) δ = 7.39-6.10 (m), 5.36-5.11 (m), 4.73-4.28 (m), 4.18-3.87 (m), 3.08-2.77 (m), 2.37-0.98 (m).
GPC: (PS calibration) $M_n = 1.6*10^4$, Đ = 1.7.

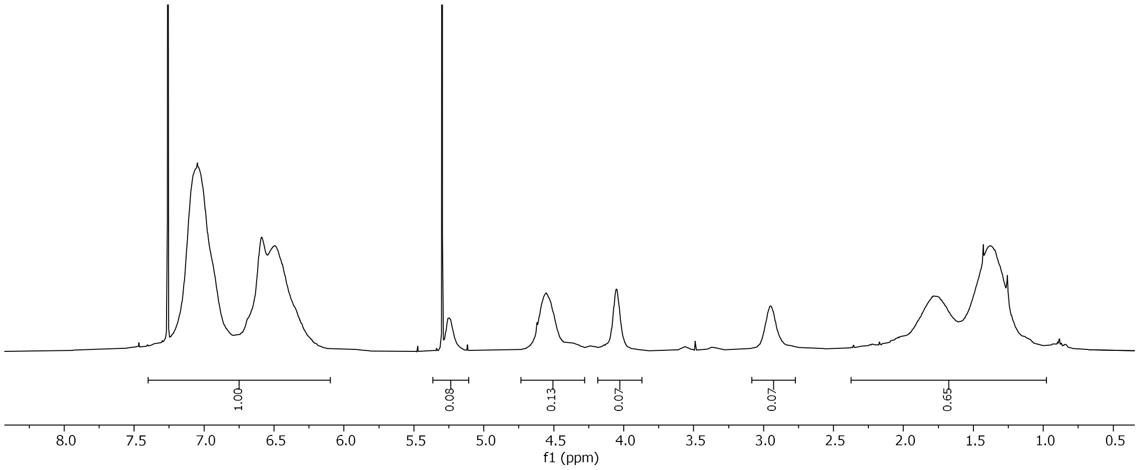

Supplementary figure S17 ^{1}H-NMR of **10**.

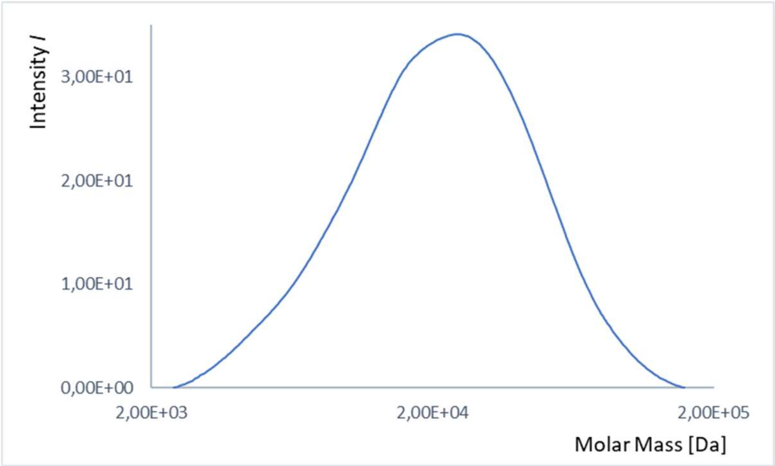

Supplementary figure S18 GPC-elugram of **10** vs PS calibration.

11

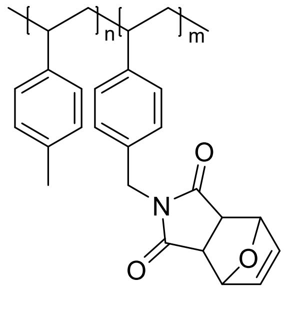

This reaction was carried out in 5 different mixing ratios (4-methylstyrene:**5** = 4:1; 5:1; 6:1; 8:1; 10:1). 15 mmol of the mixtures of 4-methylstyrene and **5** were dissolved in toluene (10 mL each). The reaction solutions of the 6:1, 8:1 and 10:1 mixtures were flushed with argon at room temperature for 30 min. The reaction solutions of the 4:1 and 5:1 batches were heated to 60 °C and flushed with argon for 30 min. All reaction mixtures were heated to 60 °C and AIBN (3 mg) in toluene (1 mL) was added. The mixtures were stirred for exactly 1 h at 60 °C to keep the conversion below 10%. The solutions were

poured into an excess of ethanol. A colourless solid precipitated, which was sedimented using a centrifuge. The solution was decanted and the solid was washed with ethanol. The solid was then dissolved twice in a small amount of DCM, precipitated again by adding an excess of ethanol and isolated as described before. **11** was obtained as a colourless solid.
^{1}H-NMR (400.1 MHz, $CDCl_3$, 298 K, ppm) δ = 7.53-5.87 (m), 5.43-5.14 (m), 4.82-4.40 (m), 3.05-0.70 (m).

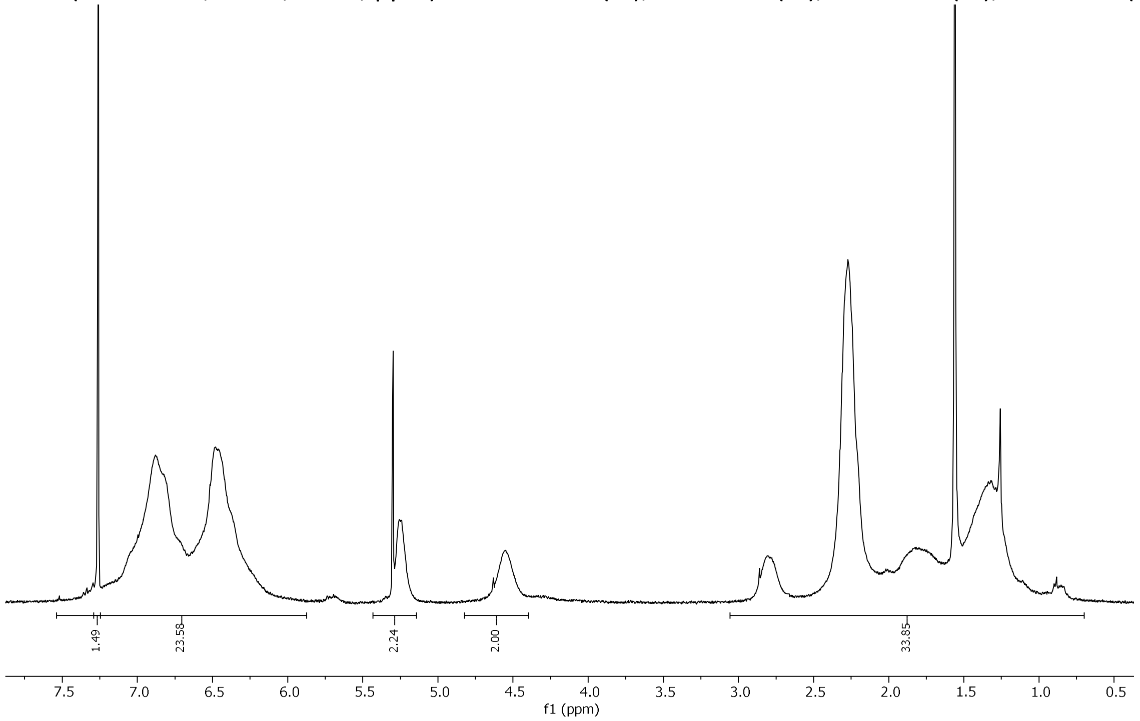

Supplementary figure S19 ^{1}H-NMR of **11** reaction batch 5:1 as an example for the reaction series.

The copolymer composition was determined by ^{1}H-NMR, comparing the integrals of the signal at around 4.5 ppm with the integrals of the aromatic region. The results as well as the monomer feed ratios are shown in **Table 1**. A plot of F^2/f vs F(f-1)/f give the values r_1 and r_2 as the slope and the *y*-intercept (**Figure 20**).

Supplementary table S2 Feed ratio 1:f and copolymer composition 1:F of different batches of the **5**/4-methylstyrene copolymerisation and resulting F^2/f and F(f-1)/f values.

Feed ratio **5**:4-Methylstyrene	Copolymer composition **5**:4-Methylstyrene	F^2/f	F(f-1)/f
1 : 4.029	1 : 3.269	2.65171158	2.4573317
1 : 5.000	1 : 4.088	3.3423488	3.2704
1 : 6.069	1 : 4.829	3.84283053	4.03356759
1 : 8.083	1 : 6.327	4.95279721	5.54442133
1 : 10.06	1 : 8.207	6.6949867	7.39101471

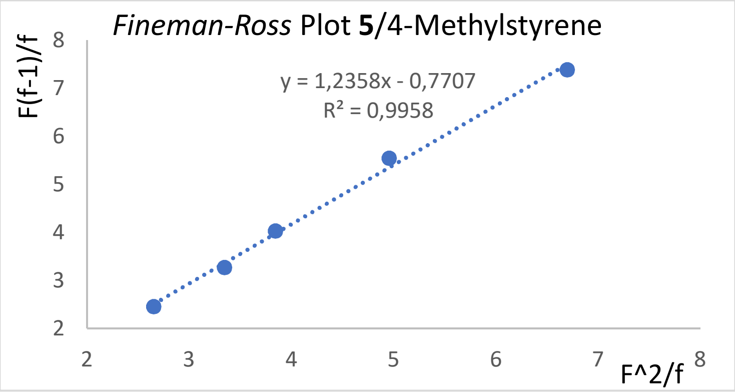

Supplementary figure S20 *Fineman-Ross*-plot for the copolymerisation of **5** with 4-methylstyrene.

12

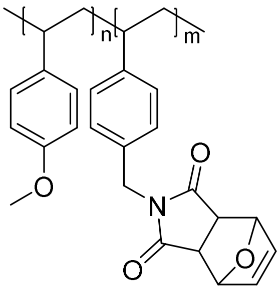

This reaction was carried out in 7 different mixing ratios (4-methoxystyrene:**5** = 4:1; 5:1; 6:1; 8:1; 10:1; 12:1; 15:1). 15 mmol of the mixtures of 4-methoxystyrene and **5** were dissolved in toluene (10 mL each). The reaction solutions were heated to 60 °C and flushed with argon for 30 min. The reaction mixtures were then heated to 65 °C and AIBN (5 mg) in toluene (1 mL) was added. The mixtures were stirred for exactly 1 h 45 min at 65 °C to keep the conversion below 10%. The solutions were then poured into an excess of ethanol. A colourless solid precipitated, which was sedimented using a centrifuge. The solution was decanted and the solid was washed with ethanol. The solid was then dissolved twice in a small amount of DCM, precipitated again by adding an excess of ethanol and isolated as described before. **12** was obtained as a colourless solid.

^{1}H-NMR (400.1 MHz, Acetone, 298 K, ppm) δ = 8.25-5.71 (m), 5.48-4.97 (m), 4.93-4.39 (m), 4.11-3.25 (m), 3.17-2.86 (m), 2.62-0.33 (m).

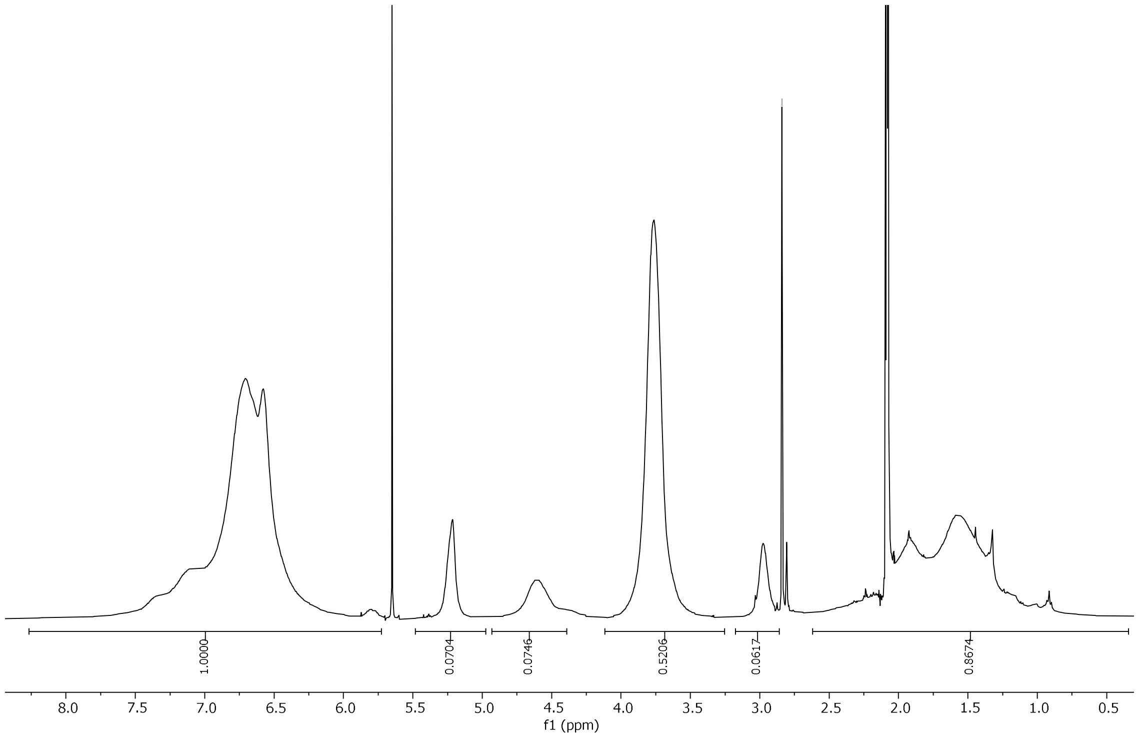

Supplementary figure S21 ^{1}H-NMR of **12** reaction batch 8:1 as an example for the reaction series.

The copolymer composition was determined by ^{1}H-NMR, comparing the integrals of the signal at around 4.5 ppm with the integrals of the aromatic region. The results as well as the monomer feed ratios are shown in **Table 2**. A plot of F^2/f vs F(f-1)/f give the values r_1 and r_2 as the slope and the *y*-intercept (**Figure 22**).

Supplementary table S3 Feed ratio 1:f and copolymer composition 1:F of different batches of the **5**/4-methoxystyrene copolymerisation and resulting F^2/f and F(f-1)/f values.

Monomer ratio **5**:4-Methoxystyrene starting mixture	Monomer ratio **M1**:4-Methoxystyrene in polymer	F^2/f	F(f-1)/f
1 : 3.999	1 : 2.694	1.814896	2.020350
1 : 4.999	1 : 3.463	2.399143	2.770537
1 : 6.032	1 : 4.246	2.988854	3.542194
1 : 7.995	1 : 5.257	3.456467	4.599221
1 : 10.02	1 : 6.560	4.296940	5.905144
1 : 11.98	1 : 7.677	4.921031	7.036261
1 : 14.99	1 : 10.35	7.148775	9.659910

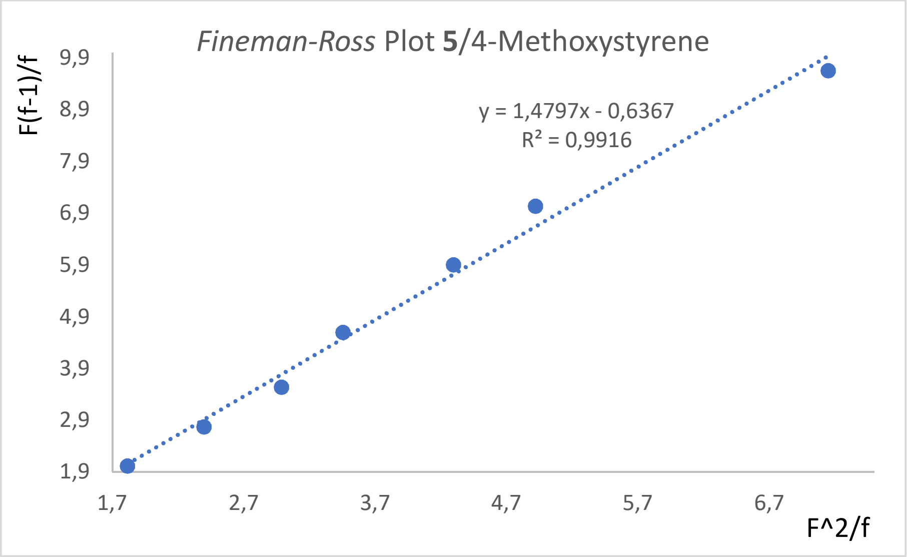

Supplementary figure S22 *Fineman-Ross*-plot for the copolymerisation of **5** with 4-methoxystyrene.

Crosslinking of 7 and 8

7 (204.6 mg, 0.435 mmol, 1.00 eq) was dissolved in toluene (5 mL). Separately, **8** (80.7 mg, 0.435 mmol, 1.00 eq) was added to toluene (5 mL) and heated to 65 °C until a clear solution was obtained. 2.5 mL of each of the two solutions were combined and shaken vigorously. A pipette of the mixture was applied to a slide, which was dried at 80 °C overnight. A slightly orange, homogeneous but brittle polymer film was obtained. The film of the cross-linked polymer curled slightly in water over a period of 15 min, but remained stable. In DCM, the film was initially stable, but slowly crumbled after a few minutes without dissolving. No healing of small surface defects at 150 °C could be observed.

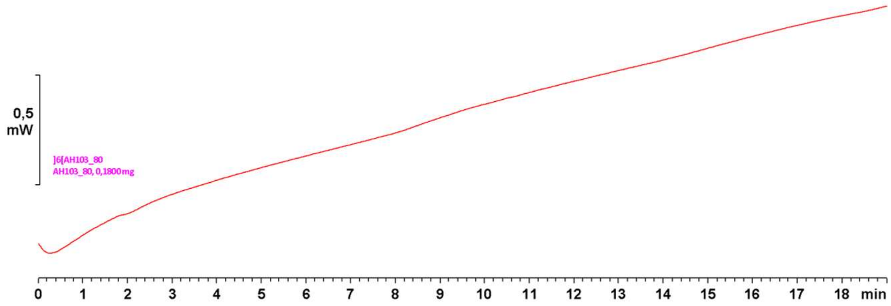

Supplementary figure S23 DSC-Measurement of crosslinked polymer. The glass transition observed for **8** is no longer visible.

Crosslinking of 7 and 9

7 (121.0 mg, 0.290 mmol, 1.00 eq) was dissolved in toluene (5 mL). Separately, **9** (221.6 mg, 0.292 mmol, 1.01 eq) was dissolved in toluene (5 mL). 2.5 mL of each of the two solutions were combined and shaken vigorously. A pipette of the mixture was applied to a slide, which was dried at 80 °C overnight. A homogenous but opaque film was formed. The film of the cross-linked polymer was stable in water over a period of 15 min. In DCM, the film was initially stable, but slowly crumbled after a few minutes without dissolving. A small scratch was made in the surface and the film was heated to 150 °C. After 24 h, the scratches had almost completely disappeared but the film obtained a brownish-yellow color which did not disappear even after cooling.
